# What Mathematical Models Are Accurate for Prescribing Aerobic Exercise in Women with Fibromyalgia?

**DOI:** 10.3390/biology11050704

**Published:** 2022-05-04

**Authors:** Santos Villafaina, Clarissa Biehl-Printes, José A. Parraca, Fabiane de Oliveira Brauner, Pablo Tomas-Carus

**Affiliations:** 1 Facultad de Ciencias del Deporte, Universidad de Extremadura, Avenida de la Universidad s/n, 10003 Caceres, Spain; 2 Departamento de Desporto e Saúde, Escola de Saúde e Desenvolvimento Humano, Universidade de Évora, 7004-516 Evora, Portugal; jparraca@uevora.pt (J.A.P.); ptc@uevora.pt (P.T.-C.); 3 Instituto de Geriatria e Gerontologia, Pontifícia Universidade Católica do Rio Grande do Sul, Porto Alegre 6681, Brazil; clarissabiehlprintes@gmail.com (C.B.-P.); fabiane.oliveira@edu.pucrs.br (F.d.O.B.); 4 Comprehensive Health Research Centre (CHRC), University of Évora, 7005 Evora, Portugal

**Keywords:** heart rate, chronic pain, physical activity, cardiopulmonary exercise testing, predictive equations

## Abstract

**Simple Summary:**

Intensity prescription for cardiorespiratory exercises is crucial for achieving health/fitness benefits. However, not all of the population can access a cardiopulmonary exercise test, either for economic reasons or location resources, to determine their ventilatory thresholds. Therefore, different mathematical models can predict the intensity based on the maximum or reserve heart rate. Exercise prescription guidelines indicate that people with fibromyalgia should exercise at 60% of their VO_2_max. However, people with fibromyalgia suffer from dysautonomia, which could lead to chronotropic incompetence, the inability to increase heart rate with increasing exercise intensities. Therefore, this study aimed to investigate the relationship and level of agreement between different mathematical models and the heart rate obtained from a cardiopulmonary exercise test at their ventilatory threshold 1. The results showed that the well-known “220 − age” at 76% and the mathematical model designed for people with fibromyalgia “209 − 0.85 × age” at 76% showed a significant level of agreement. However, Tanaka and Karvonen’s formula did not show a significant level of agreement. Thus, the “220 − age” at 76% and “209 − 0.85 × age” at 76% can be used in people with FM to prescribe aerobic exercise.

**Abstract:**

Objectives: This article aims to verify the agreement between the standard method to determine the heart rate achieved in the ventilatory threshold 1 in the cardiopulmonary exercise testing (VT1) and the mathematical models with exercise intensities suggested by the literature in order to check the most precise for fibromyalgia (FM) patients. Methods: Seventeen women with FM were included in this study. The VT1 was used as the standard method to compare four mathematical models applied in the literature to calculate the exercise intensity in FM patients: the well-known “220 − age” at 76%, Tanaka predictive equation “208 − 0.7 × age” at 76%, the FM model HRMax “209 – 0.85 × age” at 76%, and Karvonen Formula at 60%. Bland–Altman analysis and correlation analyses were used to explore agreement and correlation between the standard method and the mathematical models. Results: Significant correlations between the heart rate at the VT1 and the four mathematical estimation models were observed. However, the Bland-Altman analysis only showed agreement between VT1 and “220 − age” (bias = −114.83 + 0.868 × *x*; 95% LOA = −114.83 + 0.868 × *x* + 1.96 × 7.46 to −114.83 + 0.868 × *x* − 1.96 × 7.46, where *x* is the average between the heart rate obtained in the CPET at VT1 and “220 − age”, in this case 129.15; *p* = 0.519) and “209 − 0.85 × age”(bias = −129.58 + 1.024 × *x*; 95% LOA = −129.58 + 1.024 × *x* + 1.96 × 6.619 to −129.58 + 1.024 × *x* − 1.96 × 6.619, where *x* is the average between the heart rate obtained in the CPET at VT1 and “209 − 0.85 × age”, in this case 127.30; *p* = 0.403). Conclusions: The well-known predictive equation “220 − age” and the FM model HRMax (“209 − 0.85 × age”) showed agreement with the standard method (VT1), revealing that it is a precise model to calculate the exercise intensity in sedentary FM patients. However, proportional bias has been detected in all the mathematical models, with a higher heart rate obtained in CPET than obtained in the mathematical model. The chronotropic incompetence observed in people with FM (inability to increase heart rate with increasing exercise intensities) could explain why methods that tend to underestimate the HRmax in the general population fit better in this population.

## 1. Introduction

Fibromyalgia (FM) is characterized by generalized chronic pain, fatigue, or sleep disorders, among other symptoms [1]. These symptoms are exacerbated by physical inactivity, negatively affecting the activities of daily living and the quality of life of people with FM [2,3,4]. However, according to the American Heart Association (AHA), most people with FM are below the average levels of physical conditioning. Consequently, the maximal oxygen uptake and the anaerobic threshold decrease while the pain or fatigue increases [5,6]. Thus, aerobic exercise and strength training have been strongly recommended to keep a healthy functional capacity for people with FM [7,8].

Intensity prescription for cardiorespiratory exercises is crucial for achieving health/fitness benefits. According to the overload principle of training, the exercise below a minimum intensity or threshold will not cause physiological adaptation [9]. However, this minimum threshold varies depending on different factors such as age, health status, genetics, or habitual physical activity, among others [9,10,11]. Previous articles suggested that people with FM should perform activities above the ventilatory threshold 1 (VT1) [6,12,13]. This threshold can be obtained by analyzing ventilatory parameters or lactate values in a cardiopulmonary exercise test (CPET) using a maximal or submaximal exercise incremental protocol. In this line, guidelines for exercise prescription in people with FM suggested aerobic training [7] between 40 to 60% heart rate reserve (HRR) or VO_2_ reserve [14]; or 64 to 76% maximal heart rate (HRmax) [9,14].

However, not all of the population can access a CEPT, either for economic reasons, knowledge, or location resources. Thus, from a pedagogical perspective, Garber, Blissmer, Deschenes, Franklin, Lamonte, Lee, Nieman, and Swain [9] recommended the application of mathematical models (formulas) to calculate the target heart rate zone and predict the maximum heart rate. Among those mathematical models are those that predict HRR and HRmax. Fox 3rd, et al. [15] proposed the well-known formula to predict the HRmax (HRmax = 220 − age) [15,16]. Although there is a discussion about this formula, it has been described as one of the most used due to its practical application and simplicity [17,18]. On the other hand, studies based on meta-analysis reported that “HRmax = 208 − 0.7 × age” is a more reliable option to predict HRmax [19,20]. However, people with FM show reduced cardiorespiratory fitness [6,12,21] as well as autonomic dysfunction (dysautonomia) [13,22,23]. Dysautonomia of people with FM could lead to chronotropic incompetence, the inability to increase heart rate with increasing exercise intensities [24]. Taking into account these problems, Lemos, Valim, Zandonade, and Natour [13] developed a specific formula to calculate the HRmax of people with FM (HRmax = 209 − 0.85 × age), showing a correlation to “220 − age” and “HRmax = 208 − 0.7 × age”. However, due to the dysautonomia in people with FM, another interesting formula is the Karvonen formula. This formula (HRB + % intensity desired [HRmax – HRB]) has been recommended for people with cardiac diseases and athletes due to the accurate characteristics of the formula (using the percentage of HRR to determine the exercise intensity) [25].

Several methods can be used to calculate exercise intensity. As previously mentioned, intensity for people with FM should be higher than VT1 in order to cause physiological adaptation. However, there are people with FM or physical trainers who could not conduct a CEPT prior to an exercise intervention. Therefore, a mathematical model to estimate the intensity of exercise is recommended. However, to the best of our knowledge, any previous studies have studied the agreement between these two recommendations [6,7,12,13,14]: (1) exercising above VT1; and (2) exercising at 60% HRR or 76% HRmax. Therefore, the aim of this study was to assess the level of agreement between the HR at VT1 and the predicted HR at which people with FM would be at 60% HRR or 76% HRmax. The results would be relevant due to the dysautonomia detected in this population that could cause predictive mathematical models to underestimate or underestimate the optimal intensity to produce physiological adaptations.

## 2. Materials and Methods

### 2.1. Participants

Eighteen women with FM participated in this cross-sectional study. Participants were recruited by a Local Fibromyalgia Association (Myos, Lisbon, Portugal). Participants were included if they fulfilled the following inclusion criteria: (1) were women between 35 and 55 years old and (2) had FM diagnosis according to the American College Rheumatology (ACR) criteria (Wolfe et al., 2010). However, participants were excluded if: (1) had a condition where exercise was contraindicated, (2) had been enrolled in physical therapy in the last six months and (3) if they had a body mass index equal to or greater than 30 (kg/m^2^). One was excluded from the eighteen women recruited after conducting the CPET since the values were not correctly registered. Thus, seventeen women with FM were analyzed.

Procedures were approved by the Biomedical Ethics Committee from Cordoba University (approval number: 10396/7269), following the updates of the Helsinki Declaration.

### 2.2. Procedures

The main socio-demographic characteristics and anthropometric data (weight and height) were collected. Furthermore, participants were assessed through a CPET to obtain the VT1. In addition, four mathematical models were used to calculate 60% HRR or 76% HRmax, which was the intensity recommended by exercise prescription guidelines for people with FM [7,14].

*Cardiopulmonary exercise test(CPET)*. All participants performed a CPET using a maximal exercise incremental protocol in a cycle ergometer (JAEGER, 0 to 600 Watt) in order to determine the VT1. The protocol contained: (1) a 3 min warm-up; (2) the incremental test started at 20 watts; (3) each minute, the load was increased by 10 watts. HR and respiratory gases (Oxycon Pro-JAEGER, Hoechberg, Germany) were monitored during the protocol as well as during recovery (Medizintechnik System mit Erich Jaeger Gmbh^®^, Wuerzburg, Germany). Once the CPET was performed, VT1 was detected using Skinner’s three-phase model [26]. Participants were encouraged to report dizziness, confusion, or weakness during the CPET. If any of the participants manifested any of these symptoms, the CPET would be stopped, and blood pressure monitored. However, none of the participants reported any adverse events.

*Mathematical Models.* The following formulas were used to predict the HR at VT1: (1) “220 − age” at 76% HRmax [15,16]; (2) “208 − 0.7 × age” at 76% HRmax [20]; (3) “209 − 0.85 × age” at 76% HRmax) [13], and (4) “(HRB + 60% [HRMax – HRB])” at 60% HRR [25].

### 2.3. Statistical Analysis

Data normality was initially tested by Shapiro–Wilk test and Q-Q plots. Taking into account both the Shapiro–Wilk test and Q-Q plots, normality was assumed (see Supplementary File S1 for further details). The Bland–Altman analysis [27] was used to show agreement between the reference method VT1 obtained in a CPET and the mathematical models of training heart rate estimation. In this regard, the Bland–Altman analysis shows the dispersion graph between the difference of two variables (VT1–mathematical models) “in Y axis” and the mean of the two variables (VT1 and mathematical models) “in X axis”. To evaluate the existence of proportional bias (if the methods do not agree equally through the range of measurements), linear regressions were conducted. In this line, the differences between the methods were regressed on the average of the two methods. Following the procedure described by Bland and Altman [28], the bias (corresponding to the regression model) and the 95% limits of agreement (LOA), expressed as the regression model ± 1.96 * standard error of the residuals (the smaller range between these two limits = the better agreement) were depicted in Bland-Altman plots. The hypothesis of the bias to be or not equal to zero was tested with a *t*-test on paired samples. Pearson correlation tests were calculated between heart rate at VT1 and “220 − age” at 76%; heart rate at VT1 and “208 − 0.7 × age” at 76%; heart rate at VT1 and “209 − 0.85 × age” at 76%; and heart rate at VT1 and the Karvonen Formula at 60%. For all the tests, the significance level was defined at *p* < 0.05. All the analyses were conducted using SPSS (IBM, New York, NY, USA) (v21).

## 3. Results

Participants had a mean age of 47.5 ± 6.1 (years) and a body mass index of 25.7 ± 3.4 (kg/m^2^). Women with FM had 16.7 ± 2.0 numbers of tender points (scale 1–18) and a mean duration of symptoms of 11.5 ± 9.0 years (4.6 ± 1.5 diagnostics (years)). Participants took 2.5 ± 2.3 specific drugs (antidepressants, muscular relaxants, analgesics). Table 1 showed a poor to fair [14] aerobic fitness of people with FM (21.6 mL/min/kg). However, the physiological and cardiorespiratory parameters showed normal values (%VO_2_Max = 97.7 and %VO_2_VT1 = 69.5) [29].

**Figure 1:**
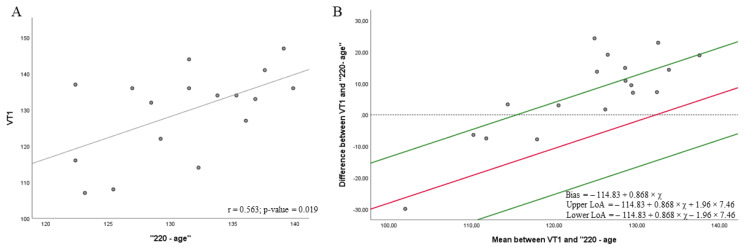
Correlations (**A**) and Bland–Altman plots (**B**) between the heart rate achieved in the VT1 in the cardiopulmonary exercise testing (CPET) and the mathematical model to predict the maximum heart rate “220 − age” at 76%.

**Figure 2:**
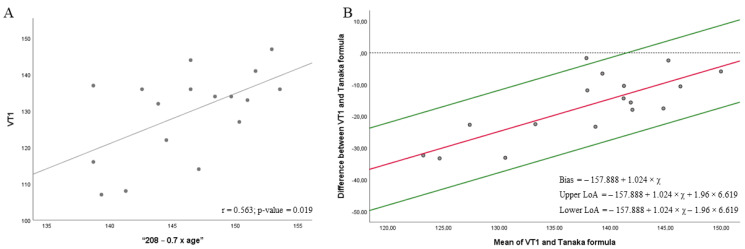
Correlations (**A**) and Bland–Altman plots (**B**) between the heart rate achieved in the VT1 in the cardiopulmonary exercise testing (CPET) and the mathematical model to predict the maximum heart rate “208 − 0.7 × age” at 76%.

**Figure 3:**
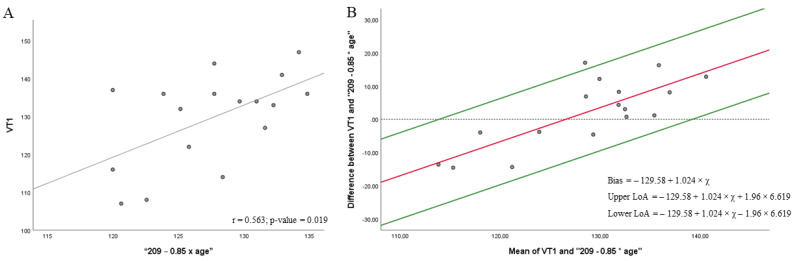
Correlations (**A**) and Bland–Altman plots (**B**) between the heart rate achieved in the VT1 in the cardiopulmonary exercise testing (CPET) and the mathematical model to predict the maximum heart rate “209 − 0.85 × age” at 76%.

**Figure 4:**
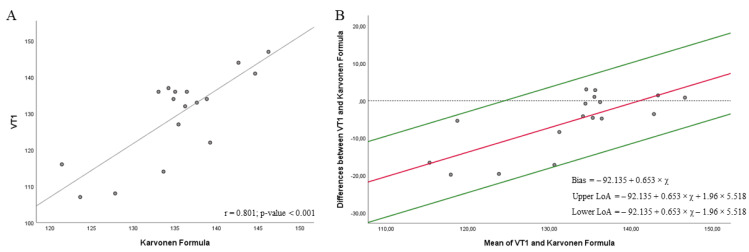
Correlations (**A**) and Bland–Altman plots (**B**) between the heart rate achieved in the VT1 in the cardiopulmonary exercise testing (CPET) and the Karvonen Formula at 60%.

Figure 1, Figure 2, Figure 3 and Figure 4 show the correlations (A) and the level of agreement (Bland–Altman plot) (B) between the gold-standard method (VT1 obtained in a CPET) and the mathematical models of HR measurement. Figure 1 shows a moderate correlation between heart rate at VT1 and “220 − age” at 76% HRmax (r = 0.563; *p* = 0.019) (Figure 1A). Regression showed a significant proportional bias (*p*-value = 0.002), and then the Bland–Altman plot was depicted according to regression results. The results from the *t*-test showed a significant agreement (Figure 1B) between heart rate at VT1 and the “220 − age” at 76% HRmax [15,16] (bias = −114.83 + 0.868 × *x*; 95% LOA = −114.83 + 0.868 × *x* + 1.96 × 7.46 to −114.83 + 0.868 × *x* − 1.96 × 7.46, where *x* is the average between the heart rate obtained in the CPET at VT1 and “220 − age”, in this case 129.15; *p* = 0.519). However, the proportional bias indicated that the heart rate obtained by CPET was higher than that obtained in the mathematical model. In addition, six measures exceeded the LOA.

Figure 2 shows a moderate correlation between the heart rate at VT1 and “208 − 0.7 × age” at 76% HRmax (r = 0.563; *p* = 0.019) (Figure 2A). Regression showed a significant proportional bias (*p*-value < 0.001), then, Bland-Altman plot was depicted according to the regression results. Results from *t*-test (Figure 2B) did not show a significant agreement between heart rate at VT1 and the “208 − 0.7 × age” formula [20] at 76% HRmax (bias = −157.888 + 1.024 × *x*; 95% LOA = −157.888 + 1.024 × *x +* 1.96 × 6.619 to −157.888 + 1.024 × *x* − 1.96 * 6.619, where *x* is the average between the heart rate obtained in the CPET at VT1 and “208 − 0.7 × age”, in this case 136.66; *p* < 0.001). The proportional bias indicated that heart rate obtained in CPET is higher than that obtained in mathematical model.

Figure 3 shows a moderate correlation between the heart rate at VT1 and “209 − 0.85 × age” at 76% HRmax (r = 0.563; *p* = 0.019) (Figure 3A). Regression showed a significant proportional bias (*p*-value < 0.001), and then the Bland–Altman plot was depicted according to the regression results. Results from the *t*-test (Figure 3B) showed significant agreement between the heart rate at VT1 and the “209 − 0.85 × age” formula at 76% HRmax [13] (bias = −129.58 + 1.024 × *x*; 95% LOA = −129.58 + 1.024 × *x* + 1.96 × 6.619 to −129.58 + 1.024 × *x* − 1.96 × 6.619, where *x* is the average between the heart rate obtained in the CPET at VT1 and “209 − 0.85 × age”, in this case 127.30; *p* = 0.403). However, the proportional bias indicated that the heart rate obtained by CPET was higher than that obtained in the mathematical model. In addition, one measure exceeded the LOA.

Figure 4 shows a strong correlation between heart rate at VT1 and the Karvonen Formula at 60% HRR (r = 0.801; *p* = <0.001) (Figure 4A). Regression showed a significant proportional bias (*p*-value = 0.001), and then the Bland–Altman plot was depicted according to the regression results. Results from the *t*-test (Figure 4B) did not show a significant agreement between heart rate at VT1 and the Karvonen Formula at 60% HRR (bias = −92.135 + 0.653 × *x*; 95% LOA = −92.135 + 0.653 × *x* + 1.96 × 5.518 to −92.135 + 0.653 × *x* − 1.96 × 5.518, where *x* is the average between the heart rate obtained in the CPET at VT1 and Karvonen Formula, in this case 131.04; *p* = 0.009) [25]. However, the proportional bias indicated that the heart rate obtained by CPET was higher than that obtained in the mathematical model.

## 4. Discussion

This study aimed to assess the level of agreement between the heart rate at VT1 obtained by CPET and the heart rate obtained by well-known mathematical models. Results showed an agreement between the “220 − age” equation and the FM model HRMax (“209 − 0.85 × age”) and the standard method (VT1) to predict the heart rate at VT1. The other two mathematical models, the Karvonen Formula at 60% of HRR and “208 − 0.7 × age”, did not show agreement, although significant correlations were observed. Thus, “220 − age” and “209 − 0.85 × age” could be considered the most precise mathematical model to determine the VT1 in people with FM. However, proportional bias has been detected in all the mathematical models, with a higher heart rate obtained in CPET than obtained in the mathematical model. Therefore, results should be taken with caution, and methods cannot be used interchangeably.

Our results are in line with those of a previous study, which reported correlations between different mathematical models and heart rate at VT1 in people with FM [13]. However, our study focused not only on the correlation analysis but also on analyzing the level of agreement between mathematical models and heart rate at VT1. In this regard, the Bland and Altman plot shows if an agreement exists when the measures of two methods report a bias around or equal to zero and an error that does not cause significant clinical impact to be replaced [27]. Thus, this statistical procedure would help to identify the agreement between the gold standard and alternative procedures, such as in our case between CPET and the mathematical models to predict VT1 [30]. According to our results, two mathematical models (“209 − 0.85 × age” and “220 − age”) showed agreement between CPET and formulas to predict VT1. 

Regarding the “209 − 0.85 × age” predictive equation, it was specifically designed for people with FM [13]. According to our results, it was the mathematical model which showed better agreement and a lower bias. However, this method also showed a proportional bias that overestimated the CPET results. This could be due to the dysautonomia suffered by people with FM [13,22,23]. In this regard, dysautonomia could make people with FM unable to increase their heart rate while increasing exercise intensity [24]. This could explain why methods that underestimate the heart rate, such as HRmax predictive equations [20,31], fit better in people with FM than others based on the HRR. In the same line, Lemos, Valim, Zandonade, and Natour [13] recommended using the 76.2% of the HRmax (using the “220 − age” equation) and 52 to 60% of HRR (using Karvonen Formula) in order to achieve strong correlations between the mathematical models and the heart rate at VT1.

As in other studies, people with FM showed proximity between VT1 and HRmax in the CPET (Table 1), which was not observed in a healthy population [6,13]. Therefore, considering that people with FM did not achieve the maximum effort, the VT1 might be considered a better fitness index than VO_2_max [6,32]. This can be due to the dysautonomia observed in people with FM that alter the sympathetic response to different stress factors such as orthostasis [22,23]. This can also explain the constant fatigue and other symptoms associated with low blood pressure, such as dizziness, confusion, and weakness [33]. 

This study has some limitations that should be acknowledged. One limitation is the relatively small size that can contribute to the decrease in statistical power to detect agreement between the methods studied. Nevertheless, this study adds relevant information in order to prescribe exercise to women with FM. However, results must be taken with caution in men with FM as well as in other people with FM who present other physical fitness levels.

## 5. Conclusions

The well-known predictive equation “220-age” and the FM model HRMax (“209 − 0.85 × age”) showed agreement between the standard method (VT1 obtained in a CPET), revealing that it is a precise model to calculate the exercise intensity in sedentary FM patients. The other mathematical models using HRmax (“208 − 0.7 × age”) or HRR, which despite showing a significant correlation, did not show agreement between the heart rate at VT1 and mathematical models as well as overestimated the heart rate at VT1. However, proportional bias has been detected in all the mathematical models, with a higher heart rate obtained in CPET than obtained in the mathematical model.

## Figures and Tables

**Table 1:** Cardiorespiratory parameters obtained in the CPET and predictive measures of heart rate of training by mathematical models of women with fibromyalgia.

Cardiorespiratory Parameters Obtained in the CPET	
Basal Heart Rate (bpm)	78.4 ± 11.6
Heart Rate at VT1 (bpm)	127.2 ± 15.4
Maximum attained Heart Rate (bpm)	145.3 ± 17.6
Maximum predicted Heart Rate 220-idade (bpm)	173 ± 6.6
VO_2_VT1 (mL/min)	1033.5 ± 175.0
VO_2_Max (mL/min)	1363.4 ± 170.0
VO_2_Max predicted (mL/min)	1486.7 ± 148.6
VO_2_Max (mL/min/kg)	21.6 ± 4.3
VO_2_VT1% predicted	69.5%
VO_2_Max% predicted	97.7%
**Mathematical Models and Predictive Measures of Heart Rate of Training**	
HRB + 60% (HRMax–HRB) [bpm]	134.8 ± 6.7
Heart Rate (220 − age) × 76% [bpm]	131 ± 5.7
Heart Rate (208 − 0.7 × age) × 76% [bpm]	146 ± 4.9
Heart Rate (209 − 0.85 × age) × 76% [bpm]	127.3 ± 4.9

Data expressed in Mean ± Standard deviation. VO_2_Max: maximum oxygen uptake; VT1: Ventilatory threshold 1; HRB: basal heart rate; HRMax: maximum heart rate; n.a.: not applicable.

## Data Availability

Data will be available upon reasonable request to corresponding author.

## Supplementary Materials

The following supporting information can be downloaded at: https://www.mdpi.com/article/10.3390/biology11050704/s1, Figure S1. Normal Q-Q plot of heart rate at ventilatory threshold 1, Figure S2. Normal Q-Q plot of “220 – age” formula, Figure S3. Normal Q-Q plot of “220 – age” formula Figure S4. Normal Q-Q plot of “208 – 0.7 × age” formula, Figure S5. Normal Q-Q plot of Karvonen formula, Table S1. Shapiro-Wilk results of heart rate achieved in the cardiopulmonary exercise test and mathematical models.
