# Supplementary material for: What Mathematical Models Are Accurate for Prescribing Aerobic Exercise in Women with Fibromyalgia?
Source: Biology (Basel). 2022 May 4;11(5):704. doi: 10.3390/biology11050704 (PMC9138585; doi:10.3390/biology11050704)
Supplement: Supplementary file 1 [file biology-11-00704-s001.zip › biology-1696833-supplementary.pdf]

Table S1. Shapiro-Wilk results of heart rate achieved in the cardiopulmonary exercise test and mathematical models.

Method	Contrast	p-value
HR at VT1	0.908	0.094
220 – age	0.943	0.353
209 – 0.85 × age	0.943	0.353
208 – 0.7 × age	0.943	0.353
Karvonen Formula	0.944	0.370

HR: Heart rate; VT1: Ventilatory threshold 1.

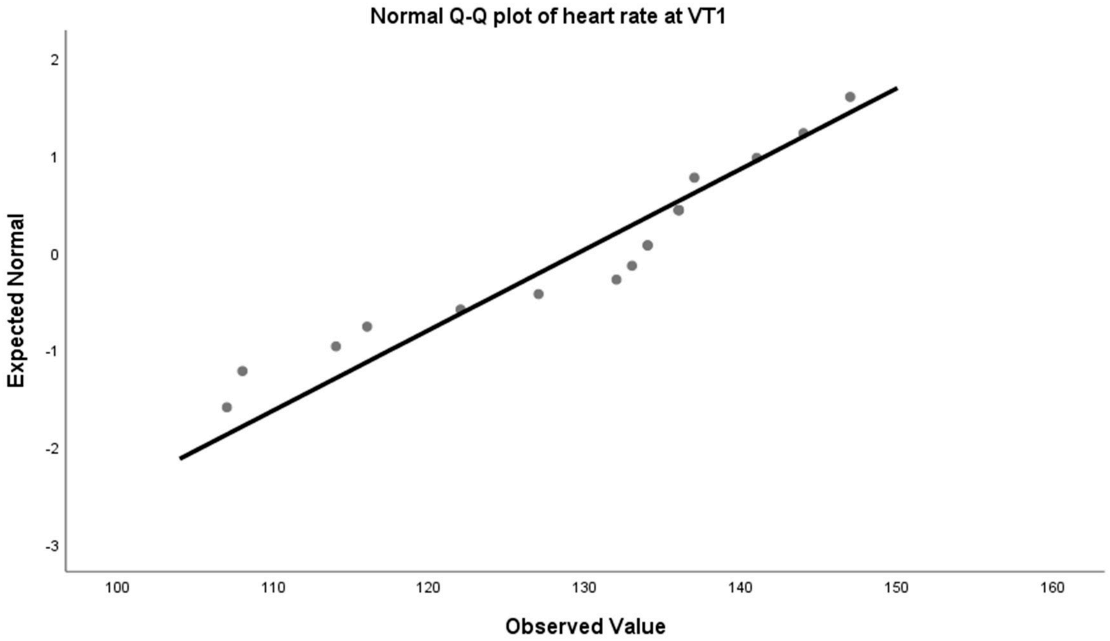

Figure S1. Normal Q-Q plot of heart rate at ventilatory threshold 1.

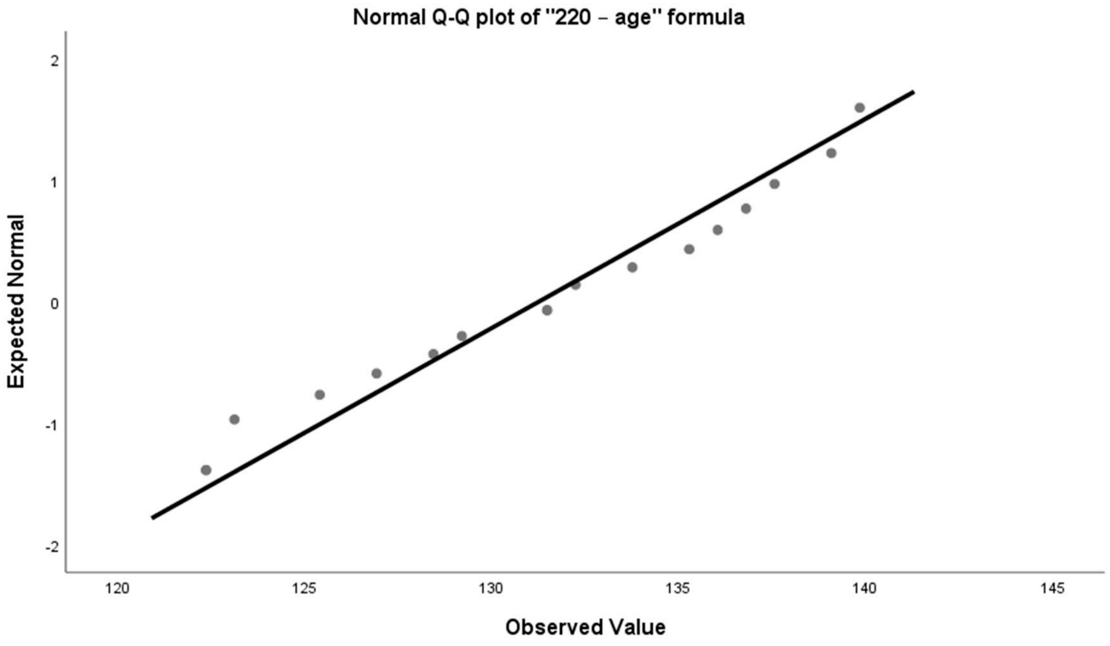

Figure S2. Normal Q-Q plot of “220 – age” formula.

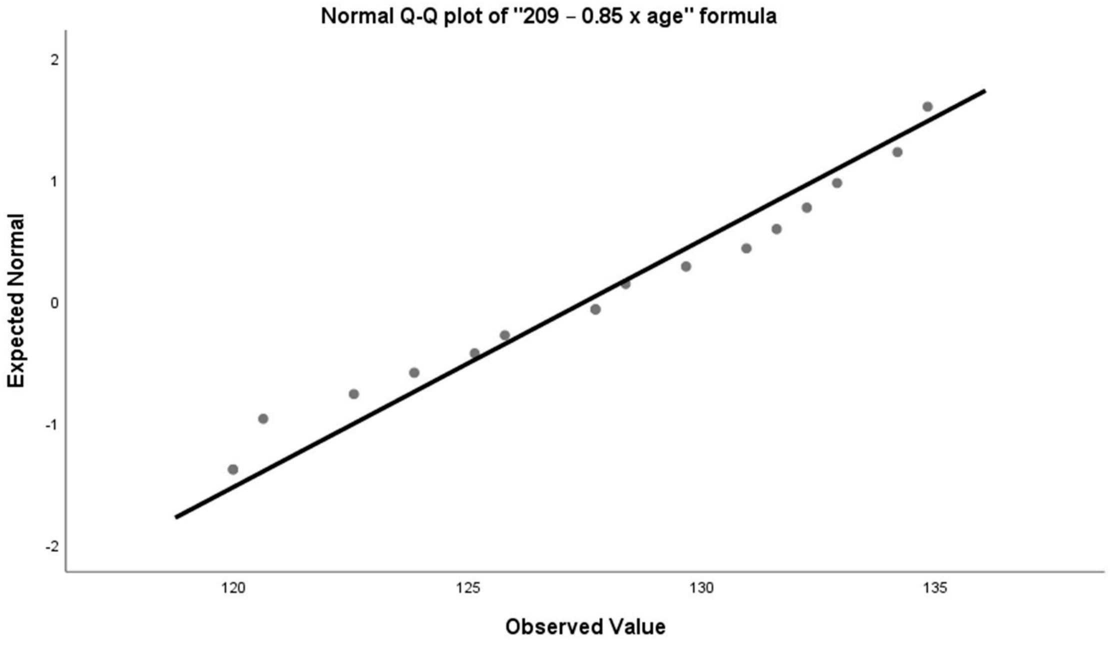

Figure S3. Normal Q-Q plot of “220 – age” formula.

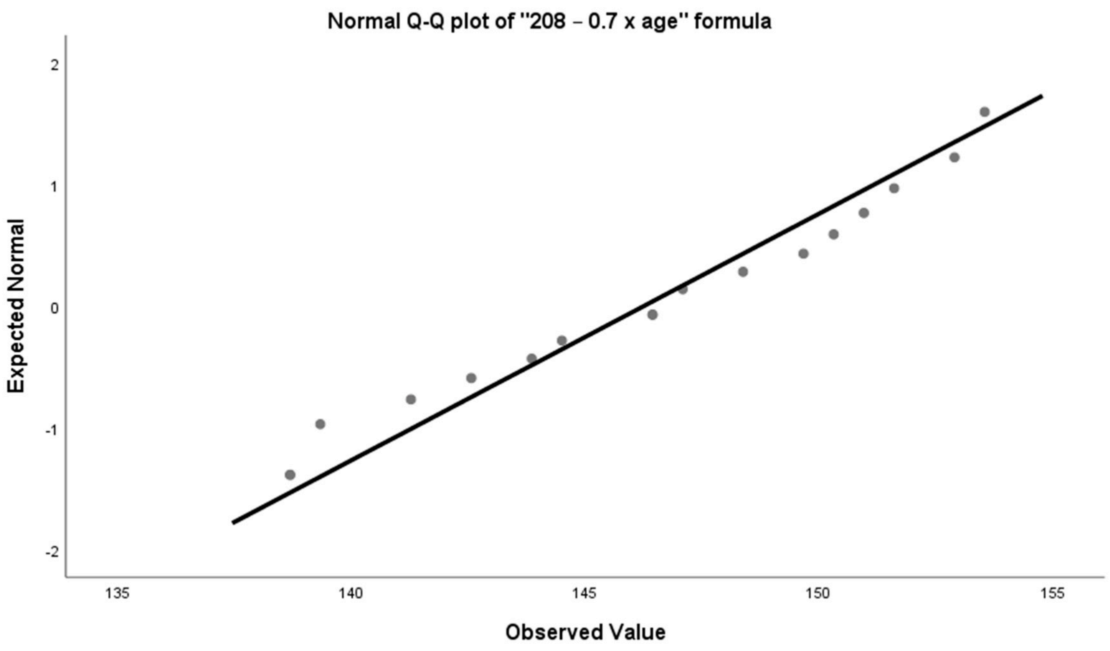

Figure S4. Normal Q-Q plot of "208 – 0.7 × age" formula.

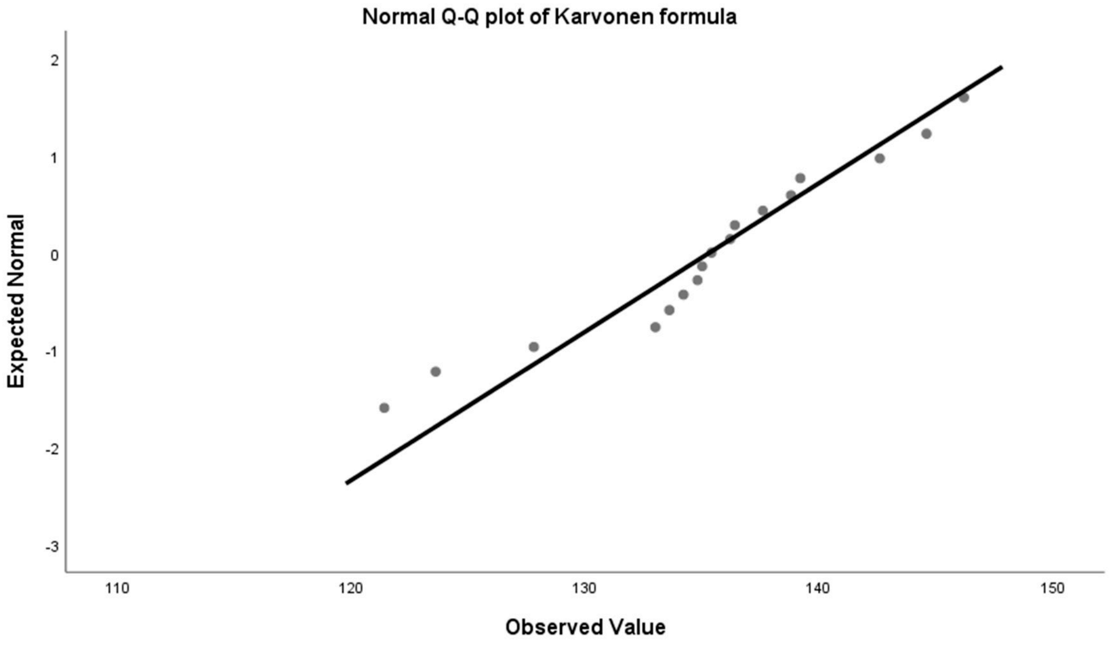

Figure S5. Normal Q-Q plot of Karvonen formula.
